# Prognostic value of supraclavicular nodes and upper abdominal nodes metastasis after definitive chemoradiotherapy for patients with thoracic esophageal squamous cell carcinoma

**DOI:** 10.18632/oncotarget.18015

**Published:** 2017-05-19

**Authors:** Xue Li, Lujun Zhao, Wencheng Zhang, Chengwen Yang, Zhen Lian, Shuai Wang, Ningbo Liu, Qingsong Pang, Ping Wang, Jinming Yu

**Affiliations:** ^1^ Department of Radiation Oncology and Key Laboratory of Cancer Prevention and Therapy, Tianjin Medical University Cancer Institute and Hospital, National Clinical Research Center for Cancer, Tianjin 300060, China; ^2^ Department of Radiation Oncology, Shandong University Affiliated Shandong Cancer Hospital and Institute, Jinan 250000, China

**Keywords:** esophageal squamous cell carcinoma, supraclavicular nodes, celiac nodes, common hepatic nodes, radiotherapy

## Abstract

The purpose of this study is to assess the prognostic value of supraclavicular nodes, left gastric nodes, celiac nodes and common hepatic nodes metastasis in esophageal squamous cell carcinoma (ESCC) treated with definitive radiotherapy. A total of 293 ESCC patients treated with radiotherapy or chemoradiotherapy entered the study. The results showed that the presence of supraclavicular nodes (χ^2^ = 0.075, *P* = 0.785) and left gastric nodes (χ^2^ = 3.603, *P* = 0.058) metastasis had no significant influence on survival, while celiac nodes (χ^2^ = 33.775, *P* < 0.001) and common hepatic nodes (χ^2^ = 42.350, *P* < 0.001) metastasis were associated with significantly shorter survival, regardless of the sites of primary tumor. Multivariate analysis showed that celiac nodes (HR: 0.457, 95% CI: 0.256-0.816; *P* = 0.008) and common hepatic nodes (HR: 0.241, 95% CI: 0.092-0.630; *P* = 0.004) metastasis were independently adverse indicator of survival in upper ESCC. While in the middle and lower ESCC, only the common hepatic nodes (middle ESCC: HR: 0.345, 95% CI: 0.161-0.738, *P* = 0.006; lower ESCC: HR: 0.377, 95% CI: 0.160-0.890, *P* = 0.026) metastasis was an independently adverse indicator of survival. In conclusion, our study demonstrated that in ESCC treated with definitive radiotherapy, both of celiac nodes and common hepatic nodes metastasis were adverse indicator of survival in upper ESCC, and common hepatic nodes metastasis were adverse indicator of survival in middle and lower ESCC. Supraclavicular nodes an left gastric nodes metastasis is not associated with patients survival in ESCC.

## INTRODUCTION

Esophageal carcinoma remains one of the most common cancer and the eighth leading cause of cancer-related deaths worldwide [1]. Esophageal squamous cell carcinoma (ESCC) has a high prevalence in East Asia, which accounts for > 90% of all types of esophageal carcinoma in China [2]. Treatment options include surgery combined with or without adjuvant chemoradiotherapy for patients with early stage ESCC, and concurrent chemoradiotherapy is the standard of care for patients with locally advanced inoperable ESCC [3]. However, the prognosis of ESCC is still poor, with a 5-year survival rate of only 17% [4].

It has been reported that both the number and location of lymph node metastasis is associated with patients survival in ESCC [5], and the 7^th^ edition of American Joint Committee on Cancer (AJCC) staging system have revised the definition of N-stage based on the number and location of lymph node metastasis [6]. In the 7^th^ edition AJCC staging system [7], as well as the recently published 8^th^ edition AJCC staging system [8], both of the supraclavicular nodes and upper abdominal nodes include left gastric nodes, celiac nodes and common hepatic nodes are regarded as regional nodes. However, this staging system is based on the results from patients treated with surgery alone, it is still unknown whether this staging system is suitable for patients treated with definitive chemoradiotherapy. Besides, the definition of regional lymph node in this staging system did not considering the site of the primary tumor. As the biological behaviour of cervical and upper thoracic ESCC is more close to head and neck squamous cell carcinoma, thus the prognosis of lymph node metastasis in patients with cervical and upper thoracic ESCC might different from patients with middle and lower thoracic ESCC [9, 10].

Thus, we conducted this study to clarify whether supraclavicular nodes, left gastric nodes, celiac nodes and common hepatic nodes have an impact on the prognosis of ESCC treated with definitive chemoradiotherapy. Besides, the impact of the site of primary tumor on the prognostic value of lymph node metastasis will also be assessed.

## RESULTS

### Patients and clinicopathological features

After the inclusion and exclusion criteria, 293 patients were included in this study. Patients and tumor characteristics are shown in Table 1. The median age of the patients was 61 years (range, 39–90 years). The median follow-up time was 17 months (range 1-77 months). Of the 293 patients, 174 patients displayed supraclavicular nodes metastasis and 118 patients displayed upper abdominal nodes metastasis. According to the 7^th^ AJCC staging system, 65 patients were N1, 121 patients were N2, and 107 patients were N3; 14 patients were stage II tumor, 248 patients were stage III tumor, and 31 patients were stage IV tumor. We also compared the baseline characteristics according to the different metastasis status of supraclavicular nodes and upper abdominal nodes (Table 1). There were no significant difference in the baseline characteristics. Besides, we found that the risk of metastasis to supraclavicular nodes or left gastric nodes was associated with the site of primary tumor, patients with upper ESCC were more prone to supraclavicular nodes metastasis while patients with lower ESCC were more prone to left gastric nodes metastasis (Table 2).

**Table 1:** Patients and tumor characteristics

Variable	Total no. of cases (% of total)	Supraclavicular nodes				Upper abdominal nodes			
		No. of positive (% of total)	No. of negative (% of total)	**χ**^2^	*P* value	No. of positive (% of total)	No. of negative (% of total)	**χ**^2^	*P* value
Sex				1.434	0.231			2.928	0.087
Male	234 (79.9)	143 (82.2)	91 (76.5)			100 (84.7)	134 (76.6)		
Female	59 (20.1)	31 (17.8)	28 (23.5)			18 (15.3)	41 (23.4)		
Age				1.007	0.316			0.136	0.712
≥70	113 (38.6)	63 (36.2)	50 (42)			44 (37.3)	69 (39.4)		
<70	180 (61.4)	111 (63.8)	69 (58)			74 (62.7)	106 (60.6)		
Tumor location				7.216	0.027*			0.562	0.755
Upper	100 (34.1)	70 (40.2)	30 (25.2)			43 (36.4)	57 (32.6)		
Middle	108 (36.9)	57 (32.8)	51 (42.9)			43 (36.4)	65 (37.1)		
Lower	85 (29)	47 (27)	38 (31.9)			32 (27.1)	53 (30.3)		
T stage				5.013	0.082			4.571	0.102
T1-2	40 (13.7)	15 (8.6)	20 (16.8)			10 (8.5)	30 (17.1)		
T3	115 (39.2)	78 (44.8)	44 (37)			48 (40.7)	67 (38.3)		
T4	138 (47.1)	81 (46.6)	55 (46.2)			60 (50.8)	78 (44.6)		
N stage				1.067	0.587			3.432	0.18
N1	65 (22.2)	35 (20.1)	30 (25.2)			20 (16.9)	45 (25.7)		
N2	121 (41.3)	74 (42.5)	47 (39.5)			50 (42.4)	71 (40.6)		
N3	107 (36.5)	65 (37.4)	42 (35.3)			48 (40.7)	59 (33.7)		
TNM stage				2.245	0.325			5.615	0.06
II	14 (4.8)	6 (3.4)	8 (6.7)			3 (2.5)	11 (6.3)		
III	248 (84.6)	148 (85.1)	101 (84.9)			107 (90.7)	141 (80.6)		
IV	31 (10.6)	20 (11.5)	10 (8.4)			8 (6.8)	23 (13.1)		
Supraclavicular nodes				283	0.000***			0.793	0.373
Positive	174 (59.4)	174 (100)	0 (0)			66 (55.9)	101 (61.2)		
Negative	119 (40.6)	0 (0)	119 (100)			52 (44.1)	64 (38.8)		
Upper abdominal nodes				0.793	0.373			283	0.000***
Positive	118 (40.3)	66 (39.5)	52 (44.8)			118 (100)	0 (0)		
Negative	175 (59.7)	101 (60.5)	64 (55.2)			0 (0)	175 (100)		
Treatment modality				0.018	0.892			3.288	0.07
Chemoradiotherapy	213 (72.7)	127 (73)	86 (72.3)			79 (66.9)	134 (76.6)		
Radiotherapy alone	80 (27.3)	47 (27)	33 (27.7)			39 (33.1)	41 (23.4)		
Radiation dose				1.794	0.18			4.529	0.033*
≥60 Gy	166 (56.7)	93 (53.4)	73 (61.3)			58 (49.2)	108 (61.7)		
<60 Gy	127 (43.3)	81 (46.6)	46 (38.7)			60 (50.8)	67 (38.3)		

*, *P* < 0.05; **, *P* < 0.01; ***, *P* < 0.001.

**Table 2:** Frequency of metastasis to supraclavicular, Left gastric, Celiac or Common hepatic node in terms of the location of primary tumor

Positive rate	Upper ESCC	Middle ESCC	Lower ESCC	X^2^	*P* value
Supraclavicular	70 (70/100)	52.8 (57/108)	55.3 (47/85)	7.216	0.027*
Left gastric	16 (16/100)	35.2 (38/108)	38.8 (33/85)	13.936	0.001***
Celiac	21 (21/100)	17.6 (19/108)	27.1 (23/85)	2.548	0.28
Common hepatic	6 (6/100)	12 (13/108)	15.3 (13/85)	4.298	0.117

*, *P* < 0.05; **, *P* < 0.01; ***, *P* < 0.001.

### Prognostic value of the location of lymph node metastasis in ESCC treated with radiotherapy

**Figure 1:**
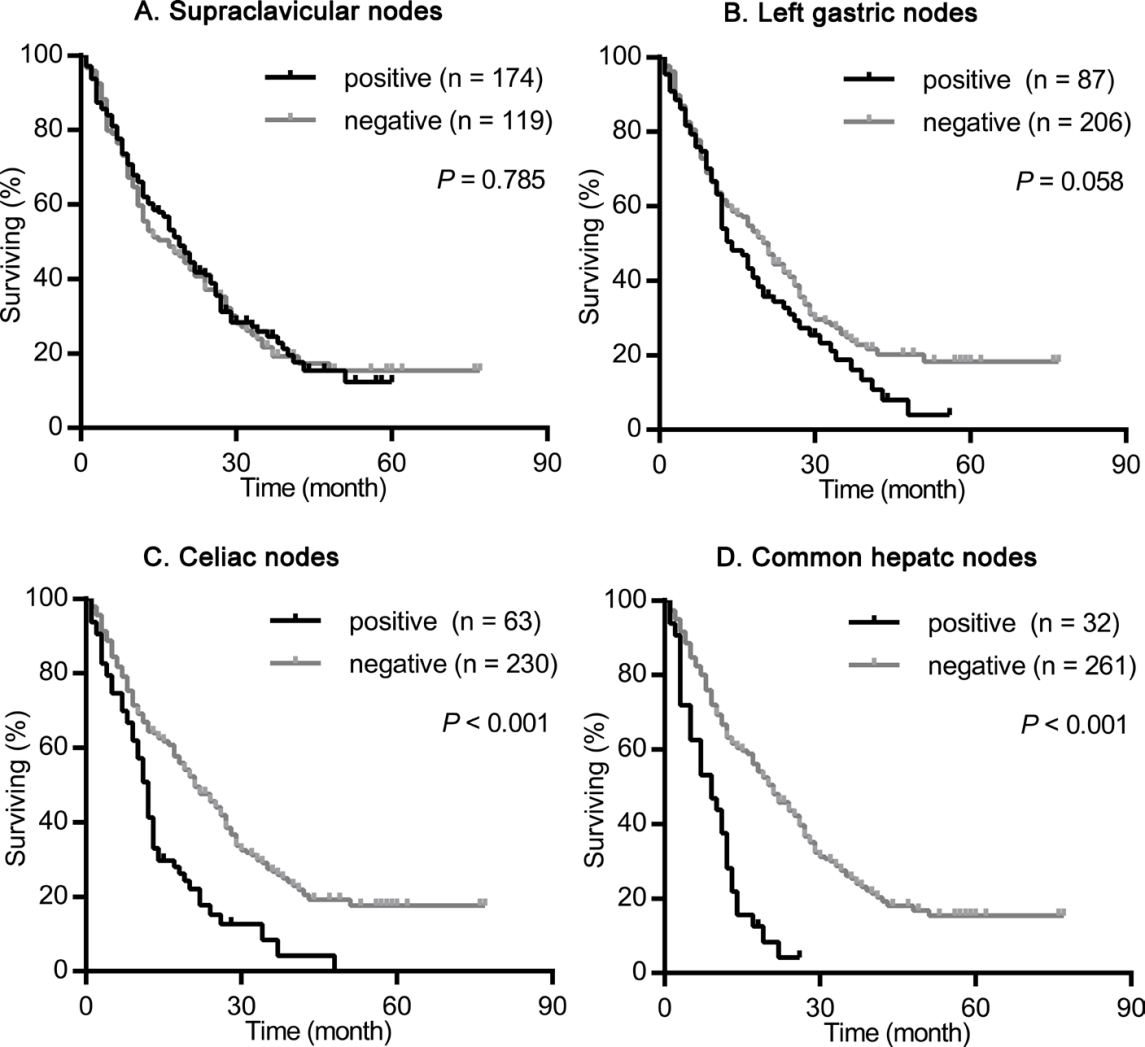
Survival analysis according the metastasis status of supraclavicular nodes, left gastric nodes, celiac nodes and common hepatic nodes in the whole population The survival curves were determined using the Kaplan-Meier method, 293 patients were included in this analysis. **(A)** Overall survival in patients with positive (174 patients) or negative (119 patients) supraclavicular nodes metastasis. **(B)** Overall survival in patients with positive (87 patients) or negative (206 patients) left gastric nodes metastasis. **(C)** Overall survival in patients with positive (63 patients) or negative (230 patients) celiac nodes metastasis. **(D)** Overall survival in patients with positive (32 patients) or negative (261 patients) common hepatic nodes metastasis.

We firstly assessed the impact of supraclavicular nodes, left gastric nodes, celiac nodes and common hepatic nodes metastasis on the survival of ESCC treated with radiotherapy. As shown in Figure 1, the presence of celiac nodes (χ^2^ = 33.775, *P* < 0.001; Figure 1C) and common hepatic nodes (χ^2^ = 42.350, *P* < 0.001; Figure 1D) metastasis were associated with significantly shorter survival compared with no evidence of metastasis. The median survival for patients with and without celiac nodes and common hepatic nodes metastasis were 11 vs. 22 months, and 7 vs. 21 months, respectively. While, the presence of supraclavicular nodes (χ^2^ = 0.075, *P* = 0.785; Figure 1A) and left gastric nodes (χ^2^ = 3.603, *P* = 0.058; Figure 1B) metastasis had no significant influence on the survival. The median survival for patients with and without supraclavicular nodes and left gastric nodes metastasis were 19 vs. 17 months, and 14 vs. 21 months, respectively.

### Impact of the site of primary tumor on the prognostic value of the location of lymph node metastasis

**Figure 2:**
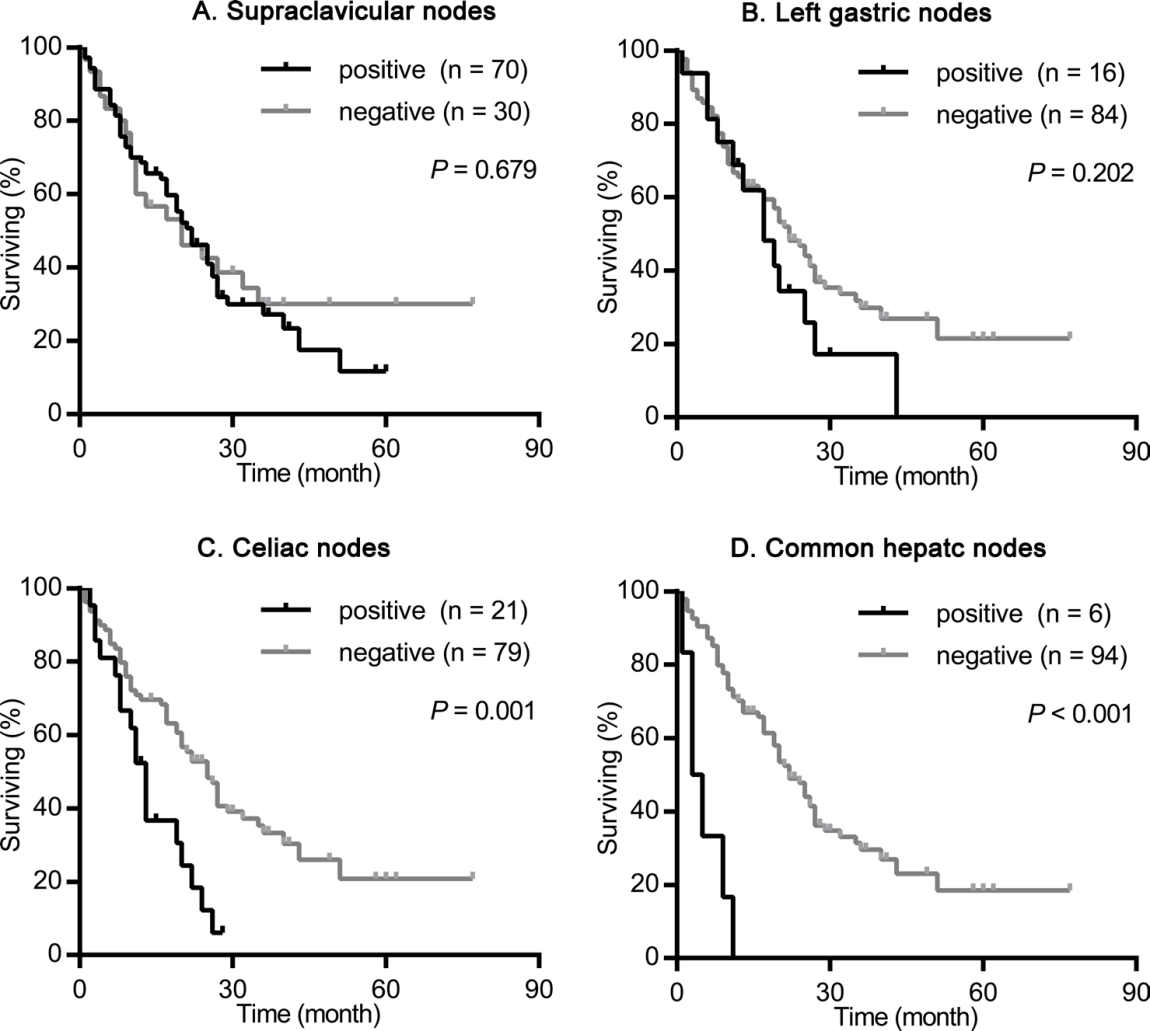
Survival analysis according the metastasis status of supraclavicular nodes, left gastric nodes, celiac nodes and common hepatic nodes in patients with upper ESCC The survival curves were determined using the Kaplan-Meier method, 100 patients were included in this analysis. **(A)** Overall survival in patients with positive (70 patients) or negative (30 patients) supraclavicular nodes metastasis. **(B)** Overall survival in patients with positive (16 patients) or negative (84 patients) left gastric nodes metastasis. **(C)** Overall survival in patients with positive (21 patients) or negative (79 patients) celiac nodes metastasis. **(D)** Overall survival in patients with positive (6 patients) or negative (94 patients) common hepatic nodes metastasis.

**Figure 3:**
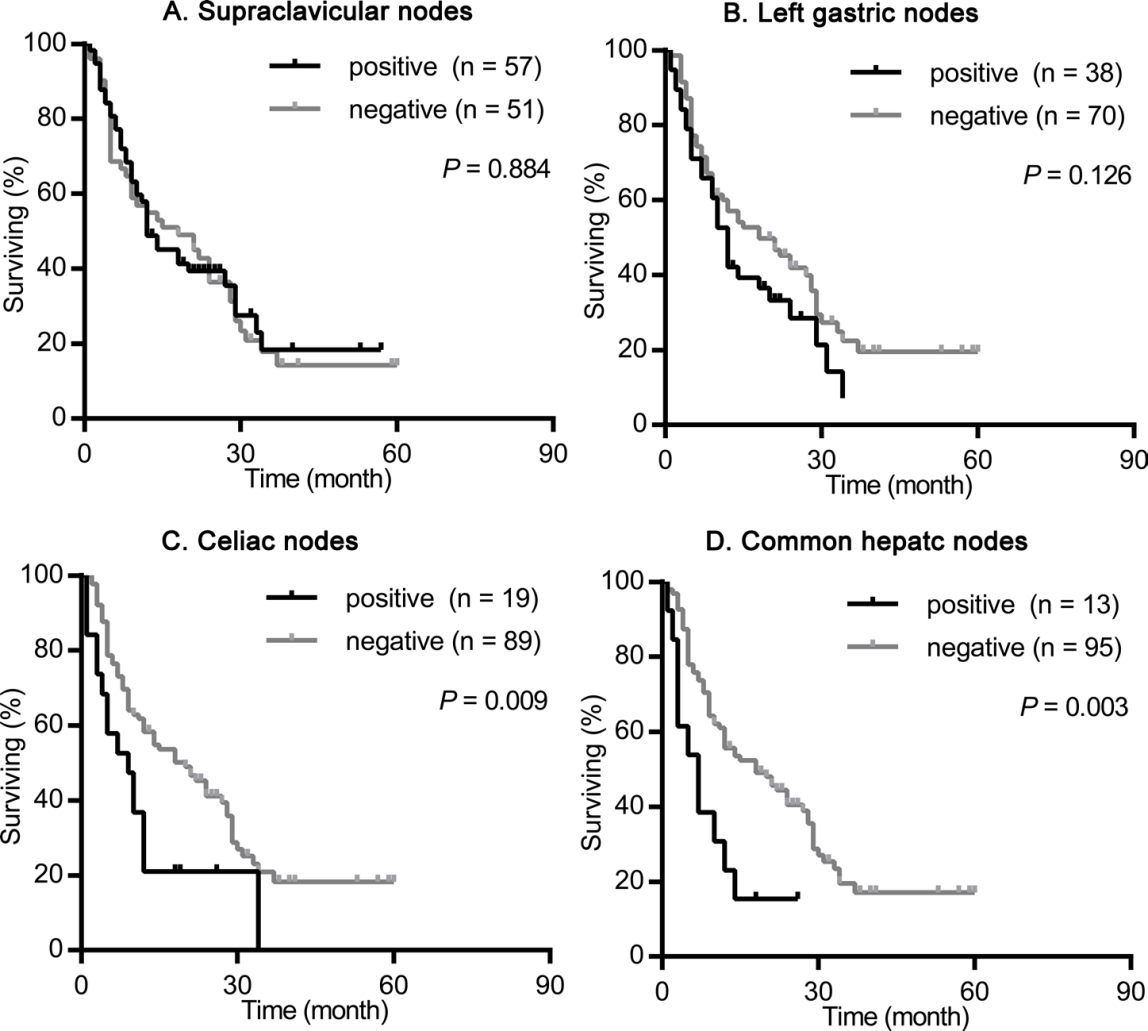
Survival analysis according the metastasis status of supraclavicular nodes, left gastric nodes, celiac nodes and common hepatic nodes in patients with middle ESCC The survival curves were determined using the Kaplan-Meier method, 108 patients were included in this analysis. **(A)** Overall survival in patients with positive (57 patients) or negative (51 patients) supraclavicular nodes metastasis. **(B)** Overall survival in patients with positive (38 patients) or negative (70 patients) left gastric nodes metastasis. **(C)** Overall survival in patients with positive (19 patients) or negative (89 patients) celiac nodes metastasis. **(D)** Overall survival in patients with positive (13 patients) or negative (95 patients) common hepatic nodes metastasis.

**Figure 4:**
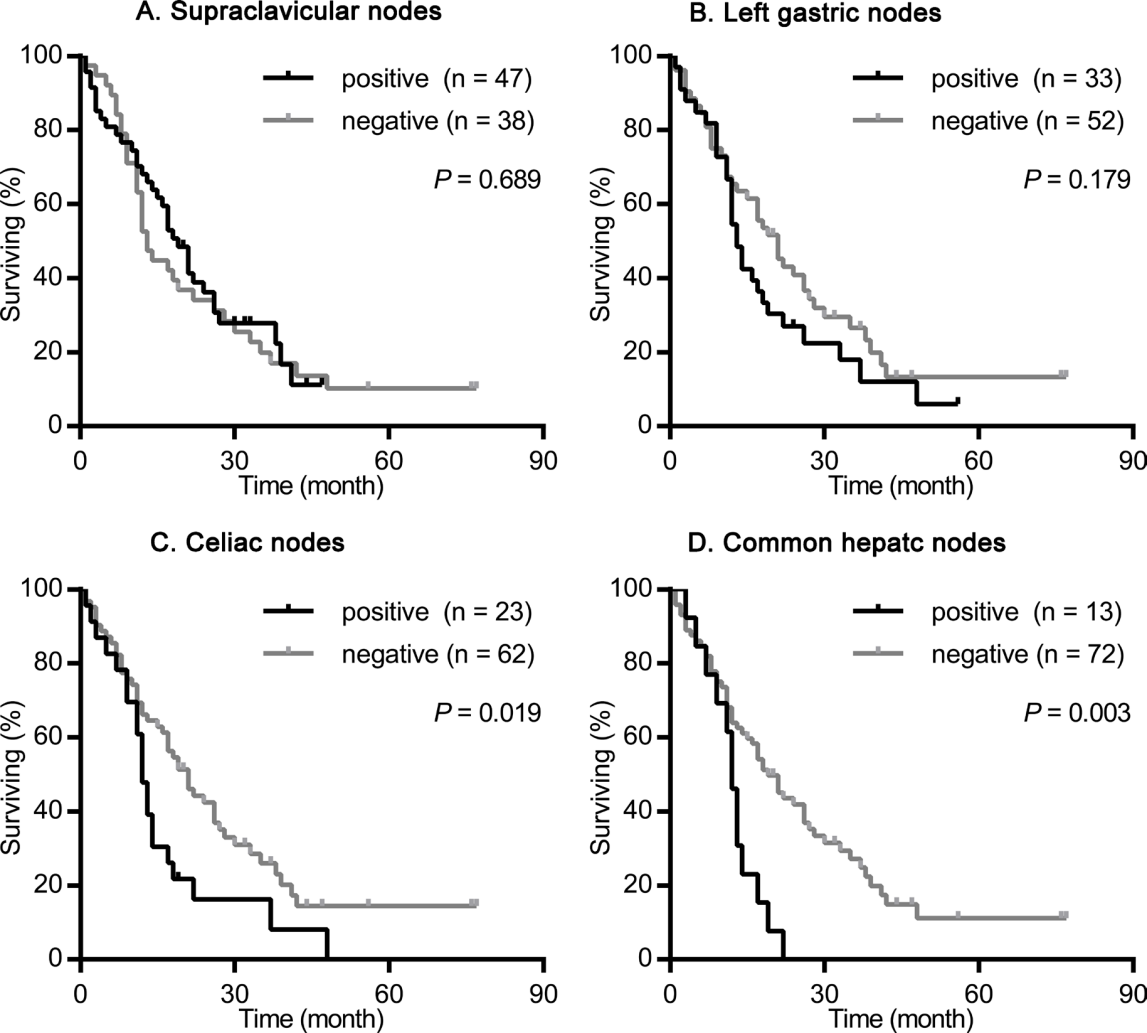
Survival analysis according the metastasis status of supraclavicular nodes, left gastric nodes, celiac nodes and common hepatic nodes in patients with lower ESCC The survival curves were determined using the Kaplan-Meier method, 85 patients were included in this analysis. **(A)** Overall survival in patients with positive (47 patients) or negative (38 patients) supraclavicular nodes metastasis. **(B)** Overall survival in patients with positive (33 patients) or negative (52 patients) left gastric nodes metastasis. **(C)** Overall survival in patients with positive (23 patients) or negative (62 patients) celiac nodes metastasis. **(D)** Overall survival in patients with positive (13 patients) or negative (72 patients) common hepatic nodes metastasis.

We nextly assessed the impact of the site of primary tumor on the prognostic value of supraclavicular nodes, left gastric nodes, celiac nodes and common hepatic nodes metastasis. As shown in Figures 2-4, the presence of celiac nodes and common hepatic nodes metastasis were associated with significantly shorter survival compared with no evidence of metastasis both in upper (for celiac nodes: χ^2^ = 15.429, *P* < 0.001, Figure 2C; for common hepatic nodes: χ^2^ = 10.712, *P* = 0.001, Figure 2D), middle (for celiac nodes: χ^2^ = 15.429, *P* < 0.001, Figure 3C; for common hepatic nodes: χ^2^ = 10.712, *P* = 0.001, Figure 3D) and lower ESCC (for celiac nodes: χ^2^ = 15.429, *P* < 0.001, Figure 4C; for common hepatic nodes: χ^2^ = 10.712, *P* = 0.001, Figure 4D). While, the presence of supraclavicular nodes and left gastric nodes metastasis had no significant influence on survival neither in upper (for supraclavicular nodes: χ^2^ = 0.171, *P* = 0.679, Figure 2A; for left gastric nodes: χ^2^ = 1.628, *P* = 0.202, Figure 2B), middle (for supraclavicular nodes: χ^2^ = 0.021, *P* = 0.885, Figure 3A; for left gastric nodes: χ^2^ = 2.346, *P* = 0.126, Figure 3B), nor lower ESCC (for supraclavicular nodes: χ^2^ = 0.301, *P* = 0.583, Figure 4A; for left gastric nodes: χ^2^ = 0.044, *P* = 0.834, Figure 4B).

### Univariate and multivariate analyses of prognostic factor

Table 3 presents the results of the univariate and multivariate analysis for survival in the whole population. Univariate analysis showed that male, higher N stage, higher T stage, higher TNM stage, positive celiac nodes, positive common hepatic nodes, treated with radiotherapy alone and radiation dose < 60Gy were associated with poor survival. While, the presence of supraclavicular nodes and left gastric nodes metastasis did not significantly influenced the survival. Multivariate analysis revealed that male, higher N stage, higher T stage, higher TNM stage, positive celiac nodes, positive common hepatic nodes, treated with radiotherapy alone and radiation dose < 60Gy were independently adverse indicator of survival.

**Table 3:** Univariate and multivariate analysis in the whole population

Variable	Median survival (months)	Univariate analysis			Multivariate analysis		
		HR	95% CI	*P* value	HR	95% CI	*P* value
Sex		0.613	0.428-0.878	0.008**	0.565	0.391-0.816	0.002**
Male	16						
Female	24						
Age		1.027	0.781-1.35	0.849			
≥70	18						
<70	19						
Tumor location		1.109	0.942-1.305	0.215			
Upper	21						
Middle	14						
Lower	17						
T stage		1.908	1.552-2.345	<0.001***	1.298	1.056-1.596	0.013**
T1-2	22						
T3	15						
T4	7						
N stage		2.483	2.041-3.021	<0.001***	2.311	1.877-2.846	<0.001***
N1	41						
N2	20						
N3	9						
TNM stage		2.939	2.082-4.149	<0.001***	2.307	1.541-3.456	<0.001***
II	43						
III	19						
IV	8						
Supraclavicular nodes		1.038	0.792-1.359	0.789			
Positive	19						
Negative	17						
Left gastric nodes		0.762	0.572-1.015	0.063			
Positive	14						
Negative	21						
Celiac nodes		0.416	0.304-0.567	<0.001***	0.599	0.416-0.862	0.006**
Positive	11						
Negative	22						
Common hepatic nodes		0.291	0.194-0.435	<0.001***	0.417	0.260-0.669	<0.001***
Positive	7						
Negative	21						
Treatment modality		1.588	1.184-2.13	0.002**	1.754	1.304-2.359	<0.001***
Chemoradiotherapy	21						
Radiotherapy alone	11						
Radiation dose		1.630	1.245-2.134	<0.001***	1.388	1.058-1.820	0.018*
≥60 Gy	22						
<60 Gy	13						

*, *P* < 0.05; **, *P* < 0.01; ***, *P* < 0.001.

Table 4 presents the results of the univariate and multivariate analysis for survival in patients with upper ESCC. The results showed that both of celiac nodes and common hepatic nodes metastasis were an independently adverse indicator of survival, while supraclavicular nodes and left gastric nodes metastasis did not significantly influenced the survival.

**Table 4:** Univariate and multivariate analysis in patients with upper ESCC

Variable	Median survival (months)	Univariate analysis			Multivariate analysis		
		HR	95% CI	*P* value	HR	95% CI	*P* value
Sex		0.560	0.311-1.009	0.053			
Male	20						
Female	26						
Age		0.921	0.568-1.492	0.737			
≥70	24						
<70	20						
T stage		2.169	1.544-3.046	<0.001***	1.553	1.090-2.212	0.015*
T1-2	43						
T3	24						
T4	17						
N stage		2.256	1.617-3.149	<0.001***	1.811	1.221-2.686	0.003**
N1	43						
N2	20						
N3	11						
TNM stage		3.417	2.002-5.832	<0.001***	2.157	1.148-4.054	0.017*
II	43						
III	22						
IV	8						
Supraclavicular nodes		0.898	0.534-1.510	0.685			
Positive	22						
Negative	20						
Left gastric nodes		0.680	0.370-1.247	0.213			
Positive	17						
Negative	22						
Celiac nodes		0.413	0.235-0.724	0.002**	0.457	0.256-0.816	0.008**
Positive	13						
Negative	25						
Common hepatic nodes		0.144	0.058-0.354	<0.001***	0.241	0.092-0.630	0.004**
Positive	3						
Negative	22						
Treatment modality		1.499	0.880-2.553	0.136			
Chemoradiotherapy	22						
Radiotherapy alone	10						
Radiation dose		1.940	1.193-3.153	0.008**	1.355	0.786-2.337	0.274
≥60 Gy	26						
<60 Gy	12						

ESCC, esophageal squamous cell carcinoma; *, *P* < 0.05; **, *P* < 0.01; ***, *P* < 0.001.

Table 5 and Table 6 presents the results of the univariate and multivariate analysis for survival in patients with middle and lower ESCC. The results showed that common hepatic nodes metastasis were independently adverse indicator of survival, while supraclavicular nodes, left gastric nodes and celiac nodes metastasis did not significantly influenced the survival neither in middle nor lower ESCC.

**Table 5:** Univariate and multivariate analysis in patients with middle ESCC

Variable	Median survival (months)	Univariate analysis			Multivariate analysis		
		HR	95% CI	*P* value	HR	95% CI	*P* value
Sex		0.729	0.385-1.379	0.332			
Male	12						
Female	24						
Age		0.800	0.510-1.255	0.332			
≥70	12						
<70	21						
T stage		1.781	1.252-2.532	0.001**	1.562	1.061-2.300	0.024*
T1-2	28						
T3	24						
T4	8						
N stage		2.951	2.047-4.254	<0.001***	3.040	2.062-4.483	<0.001***
N1	45						
N2	21						
N3	9						
TNM stage		2.126	1.255-3.601	0.005**	1.474	0.767-2.833	0.244
II	44						
III	14						
IV	9						
Supraclavicular nodes		1.032	0.663-1.608	0.885			
Positive	12						
Negative	18						
Left gastric nodes		0.704	0.442-1.120	0.138			
Positive	12						
Negative	18						
Celiac nodes		0.494	0.283-0.862	0.013*	0.733	0.381-1.409	0.351
Positive	9						
Negative	20						
Common hepatic nodes		0.402	0.209-0.773	0.006**	0.345	0.161-0.738	0.006**
Positive	7						
Negative	18						
Treatment modality		1.628	1.010-2.624	0.045*	2.174	1.312-3.601	0.003**
Chemoradiotherapy	21						
Radiotherapy alone	7						
Radiation dose		1.572	1.000-2.479	0.044*	1.284	0.803-2.053	0.297
≥60 Gy	21						
<60 Gy	12						

*, *P* < 0.05; **, *P* < 0.01; ***, *P* < 0.001.

**Table 6:** Univariate and multivariate analysis in patients with lower ESCC

Variable	Median survival (months)	Univariate analysis			Multivariate analysis		
		HR	95% CI	*P* value	HR	95% CI	*P* value
Sex		0.598	0.310-1.154	0.125			
Male	17						
Female	26						
Age		1.553	0.934-2.582	0.09			
≥70	17						
<70	16						
T stage		1.060	0.705-1.594	0.778			
T1-2	27						
T3	14						
T4	17						
N stage		2.479	1.742-3.529	< 0.001***	2.106	1.447-3.064	< 0.001***
N1	33						
N2	19						
N3	9						
TNM stage		5.241	2.108-13.03	< 0.001***	4.814	1.792-12.928	0.002**
II							
III	18						
IV	7						
Supraclavicular nodes		1.102	0.679-1.787	0.695			
Positive	13						
Negative	19						
Left gastric nodes		1.052	0.648-1.710	0.837			
Positive	16						
Negative	17						
Celiac nodes		0.347	0.206-0.584	< 0.001***	0.574	0.292-1.128	0.107
Positive	11						
Negative	21						
Common hepatic nodes		0.264	0.136-0.513	< 0.001***	0.377	0.160-0.890	0.026*
Positive	11						
Negative	19						
Treatment modality		1.502	0.893-2.526	0.126			
Chemoradiotherapy	17						
Radiotherapy alone	17						
Radiation dose		1.415	0.877-2.284	0.155			
≥60 Gy	17						
<60 Gy	14						

*, *P* < 0.05; **, *P* < 0.01; ***, *P* < 0.001.

## DISCUSSION

Our study indicated that the presence of celiac nodes and common hepatic nodes metastasis were independently adverse indicator of survival in upper ESCC treated with definitive radiotherapy. While in middle and lower ESCC, only the common hepatic nodes metastasis was an independently adverse indicator of survival. The presence of supraclavicular nodes and left gastric nodes metastasis did not influence patients survival, neither in upper, middle nor lower thoracic ESCC.

Several studies reported that involvement of supraclavicular nodes metastasis commonly present good outcomes in patients treated with surgery [11–14]. A study including 86 patients reported that the presence of supraclavicular nodes had no influence on the survival of patients treated with surgery [14]. Another study including 323 patients demonstrated that supraclavicular nodes metastasis reflect the number of metastatic lymph nodes rather than distant metastasis [12]. Furthermore, a recently published data including 1156 ESCC patients indicated that regarding supraclavicular nodes as regional nodes is better in stratification of survival compared with as nonregional nodes [15]. These studies indicated that the supraclavicular nodes should be regarded as regional lymph nodes rather than nonregional lymph nodes. However, all of the studies mentioned above were conducted in patients treated with surgery, it is still unknown about the impact of supraclavicular nodes metastasis on the survival of patients treated with radiotherapy. Our study showed that in patients treated with radiotherapy, there was no significant difference in survival between patients with supraclavicular nodes metastasis and those without. The results from our study indicated that the presence of supraclavicular nodes metastasis did not further decrease patients survival compared with other regional nodes metastasis. Thus, supraclavicular nodes should be regarded as regional nodes and should not influence the performance of curative radiotherapy.

Current treatment options for ESCC patients with supraclavicular nodes metastasis include surgery and chemoradiotherapy [16]. However, there are debates about the performance of surgery for these patients. As aggressive extended lymphadenectomy, especially the three field lymphadenectomy, commonly leads to significantly enhanced perioperative morbidity [17, 18]. In contrast, several studies have demonstrated that definitive chemoradiotherapy is an effective and safety treatment option for ESCC patients with supraclavicular nodes metastasis [19–22]. Especially, a recently published data showed that intensity-modulated radiotherapy (IMRT) combined with hyperthermia is well tolerated with excellent local control in ESCC with supraclavicular node metastasis [23]. Thus, chemoradiotherapy might be an better option for patients with supraclavicular nodes metastasis, yet it is still need larger randomized controlled clinical trials to confirm it.

However, the prognostic value of the left gastric nodes, celiac nodes and common hepatic nodes metastasis in ESCC is still undefined, even these nodes are defined as regional nodes in the 7^th^ edition AJCC staging system. Several studies have been conducted to evaluate the impact of celiac nodes metastasis on the survival of ESCC treated with surgery [24–27]. A study including 1027 patients treated with surgery showed that no significant difference were found in survival between patients with celiac nodes metastasis and these with other regional nodes metastasis, they concluded that celiac nodes should be regard as regional nodes [5]. The Mayo clinic reported similar results in patients with distal esophagus or gastroesophageal junction carcinoma who were treated with definitive esophagectomy [28]. Another study including 665 patients treated with surgery concluded that the celiac nodes was not an independent adverse prognostic factor, while the common hepatic nodes was [13]. However, in patients treated with definitive radiotherapy, there have been only one study evaluated the prognostic value of celiac nodes metastasis. The study including 144 patients showed that celiac nodes metastasis is a strong predictor of poor outcome in patients with ESCC [29]. Our study found similar results. Moreover, we found that the prognostic value of upper abdominal nodes, which including left gastric nodes, celiac nodes and common hepatic nodes, was influenced by the sites of primary tumor. In upper ESCC, the presence of celiac nodes and common hepatic nodes was an independently adverse indicator of survival. While in middle and lower ESCC, only the common hepatic nodes was an independently adverse indicator of survival.

Our study also showed that the survival of patients received radiotherapy with ≥ 60Gy significantly longer than patients received < 60Gy. This was in line with several previous study [30, 31], and the regimen of concurrent chemoradiotherapy with 60Gy radiotherapy in 30 fractions is widely used in Japan [32]. Even though, the recommended standard dose were 50 or 50.4 Gy for definitive chemoradiotherapy in the NCCN esophageal cancer guidelines, which were based on the results of RTOG 9405 [33]. The results of RTOG 9405 showed that chemoradiotherapy with 64.8Gy did not increased patients survival compared to these with 50.4Gy. However, there were several potential explanations for the lack of benefit in the high-dose arm in RTOG 9405 [34]. Firstly, there was a significant prolongation in treatment duration in the high-dose arm. Secondly, the dose of 5-FU administered in the high-dose arm was significantly lower than these in standard-dose arm. In this study, the dose of chemotherapy were parallel between the ≥ 60Gy group and the < 60Gy group. Besides, the radiotherapy was delivered with 3D-CRT or IMRT in all of the patients, this would permit improved radiation dose delivery to tumor with sparing of normal tissue. However, further clinical study still need to confirm the role of high-dose radiotherapy in the treatment ESCC.

Among the limitations of this study were its retrospective nature, with the associated biases. Besides, patients included in this study were treated with chemoradiotherapy without surgery, thus, the diagnosis of positive lymph nodes are mainly depended on the image rather than the pathological assessments. Therefore, we cannot prove that all of the identified enlarged lymph nodes contain metastatic disease. Another potential weakness is the heterogeneity of the patient population in terms of age (range, 39–90 years). Moreover, a median follow-up of 17 months is rather short to precisely evaluate OS, as well as their prognostic factors. Therefore, our results should be further evaluated in other large cohorts.

In conclusion, our study demonstrated that in ESCC treated with definitive radiotherapy, both of celiac nodes and common hepatic nodes metastasis were adverse indicator of survival in upper ESCC, and common hepatic nodes metastasis were adverse indicator of survival in middle and lower ESCC. On the other hand, supraclavicular nodes an left gastric nodes metastasis is not associated with patients survival neither in upper, middle nor lower ESCC.

## MATERIALS AND METHODS

### Ethics statement

Investigation has been conducted in accordance with the ethical standards and according to the Declaration of Helsinki and according to national and international guidelines and has been approved by the authors' institutional review board.

### Patients

Between January 2008 and January 2013, 756 patients with ESCC were treated with radiotherapy alone or radiotherapy combined with chemotherapy in Tianjin Medical University Cancer Institute and Hospital. These patients had either refused surgery or were unable to undergo surgery. Inclusion criteria for patients enrolled in this study include: pathological diagnosed as ESCC, and treated with radiotherapy alone or radiotherapy combined with chemotherapy. Patients who had received surgery were excluded from this study. TNM staging was defined according to the 7^h^ edition staging system.

### Radiotherapy and chemotherapy

CT-based radiation planning and 3D conformal radiotherapy (3D-CRT) or intensity modulated radiotherapy (IMRT) were used in the patients. All patients were treated by conventional fractionation (1.8-2 Gy per fraction, one fraction per day and five fractions per week), the median dose of radiation delivered was 60 Gy (range 40–70 Gy). The gross tumor volume (GTV) included primary tumor and involved lymph nodes. The clinical target volume (CTV) included the GTV with a 3 cm margin in the craniocaudal direction and a 0.5 cm margin in the lateral and anteroposterior directions. The CTV of ESCC involving the upper third of the esophagus encompassed the right and left supraclavicular regions. In patients with unilateral supraclavicular nodes metastasis, the contralateral supraclavicular fossa was included in the CTV for prophylactic purposes. The CTV for lymph nodes included the GTV-N without an additional margin. The planning target volume (PTV) included the CTV with a 1-cm margin in the superior–inferior direction and a 0.5 cm margin in the lateral direction.

Chemotherapy was administered using regimens that mainly included cisplatin plus 5-fluorouracil and cisplatin plus docetaxel. In all, 110 patients were treated with two cycles of 60 mg/m^2^ docetaxel and 80 mg/m^2^ cisplatin delivered on days 1 and 22 of radiotherapy; 21 patients received at least four cycles of docetaxel (30 mg/m^2^) and cisplatin (35 mg/m^2^) per week; another 72 patients were treated with two cycles of 60 mg/m^2^ cisplatin administered on days 1 and 29 and 300 mg/m^2^/24h 5-fluorouracil administered on days 1–3 and days 29–31.

### Lymph node station definition

Tumor evaluation was based on esophagoscopy, barium esophagography, CT scan (chest and abdominal) and ultrasonography (neck and abdominal). The status of lymph nodes were decided according to the results of CT scan (chest and abdominal) and ultrasonography (neck and abdominal). Features supporting a consideration for clinical metastasis included: 1) Nodes with a short axis ≥ 1 cm or these in periesophageal ≥ 0.5 cm on CT scan; 2) Nodes with necrotic center or inhomogeneous enhancement, regardless of the long of the axis. The status of the primary tumor were decided according to the esophagoscopy, barium esophagography and CT scan (chest and abdominal). The results of esophagoscopy and biopsy decided the pathologic diagnosis, the barium esophagography decided the upper and lower border of the primary tumor, and the T stage were decided according to the CT scan. In the CT scan, it is difficult to distinguish T1 from T2, thus we diagnosed the T stage as T1/2, T3 and T4. Tumor location were classified according to the AJCC criteria. The cervical ESCC were tumors between upper esophageal sphincter and sternal notch, the upper thoracic ESCC were tumors between sternal notch and azygos vein, the middle thoracic ESCC were tumors between azygos vein and inferior pulmonary vein, and the lower thoracic ESCC were tumors between inferior pulmonary vein and lower esophageal sphincter. For analytical purposes, we classified the tumors into 3 groups, the upper esophageal (above the azygos vein, include the cervical and upper thoracic esophageal), the middle esophageal (between azygos vein and inferior pulmonary vein, include the middle thoracic esophageal), and the lower esophageal (between inferior pulmonary vein and lower esophageal sphincter, include the lower thoracic esophageal).

Lymph nodes were assigned a station designation according to the AJCC criteria [8]. Briefly, the supraclavicular nodes are nodes above suprasternal notch and clavicles, celiac nodes are at the base of the celiac artery, left gastric nodes are along the course of the left gastric artery, and common hepatic nodes are immediately on the proximal common hepatic artery. For analytical purposes, nodes at the upper abdominal region, including left gastric nodes, celiac nodes and common hepatic nodes, were designated as “upper abdominal nodes”.

### Statistical methods

The primary endpoint of this study was overall survival (OS). OS was calculated from the day of diagnosis to the date of death or the date of last follow-up. Survival curves were determined using the Kaplan-Meier method. Prognostic factors for OS was obtained using the log-rank test. Cox regression was used to evaluate the hazard ratios (HR) as well as the 95% confidence intervals. All prognostic factors with a *P* < 0.05 were included for a multivariate analysis using a Cox regression. The χ^2^ test was used to compare the difference of patients’ characteristics. Statistical significance was defined with a *P* value < 0.05. Statistical analyses were performed using SPSS 17.0 (SPSS, Inc., Chicago, IL, USA).

## References

[1] Torre LA, Bray F, Siegel RL, Ferlay J, Lortet-Tieulent J, Jemal A Global cancer statistics, 2012. CA Cancer J Clin.

[2] Zhang Y (2013). Epidemiology of esophageal cancer. World J Gastroenterol.

[3] Stahl M, Budach W, Meyer HJ, Cervantes A (2010). ESMO Guidelines Working Group. Esophageal cancer: Clinical Practice Guidelines for diagnosis, treatment and follow-up. Ann Oncol.

[4] Siegel R, Naishadham D, Jemal A (2013). Cancer statistics 2013. CA Cancer J Clin.

[5] Hofstetter W, Correa AM, Bekele N, Ajani JA, Phan A, Komaki RR, Liao Z, Maru D, Wu TT, Mehran RJ, Rice DC, Roth JA, Vaporciyan AA (2007). Proposed modification of nodal status in AJCC esophageal cancer staging system. Ann Thorac Surg.

[6] Kikuchi S, Futawatari N, Sakuramoto S, Katada N, Yamashita K, Shibata T, Nemoto M, Watanabe M (2011). Comparison of staging between the old (6th edition) and new (7th edition) TNM classifications in advanced gastric cancer. Anticancer Res.

[7] Rice TW, Blackstone EH, Rusch VW (2010). 7th edition of the AJCC Cancer Staging Manual: esophagus and esophagogastric junction. Ann Surg Oncol.

[8] Rice TW, Ishwaran H, Ferguson MK, Blackstone EH, Goldstraw P (2017). Cancer of the esophagus and esophagogastric junction: an eighth edition staging primer. J Thorac Oncol.

[9] Tachimori Y, Nagai Y, Kanamori N, Hokamura N, Igaki H (2011). Pattern of lymph node metastases of esophageal squamous cell carcinoma based on the anatomical lymphatic drainage system. Dis Esophagus.

[10] Merkow RP, Bilimoria KY, Keswani RN, Chung J, Sherman KL, Knab LM, Posner MC, Bentrem DJ (2014). Treatment trends, risk of lymph node metastasis, and outcomes for localized esophageal cancer. J Natl Cancer Inst.

[11] Tachimori Y, Ozawa S, Numasaki H, Matsubara H, Shinoda M, Toh Y, Udagawa H (2014). Registration Committee for Esophageal Cancer of the Japan Esophageal Society. Supraclavicular node metastasis from thoracic esophageal carcinoma: a surgical series from a Japanese multi-institutional nationwide registry of esophageal cancer. J Thorac Cardiovasc Surg.

[12] Miyata H, Yamasaki M, Miyazaki Y, Takahashi T, Kurokawa Y, Nakajima K, Takiguchi S, Mori M, Doki Y (2015). Clinical importance of supraclavicular lymph node metastasis after neoadjuvant chemotherapy for esophageal squamous cell carcinoma. Ann Surg.

[13] Yamasaki M, Miyata H, Miyazaki Y, Takahashi T, Kurokawa Y, Nakajima K, Takiguchi S, Mori M, Doki Y (2014). Evaluation of the nodal status in the 7th edition of the UICC-TNM classification for esophageal squamous cell carcinoma: proposed modifications for improved survival stratification: impact of lymph node metastases on overall survival after esophagectomy. Ann Surg Oncol.

[14] Kosugi S, Kawaguchi Y, Kanda T, Ishikawa T, Sakamoto K, Akaike H, Fujii H, Wakai T (2013). Cervical lymph node dissection for clinically submucosal carcinoma of the thoracic esophagus. Ann Surg Oncol.

[15] Zheng Y, Wang Z, Wang F, Huang Q, Liu S (2017). Proposed modifications of supraclavicular lymph node metastasis in the esophageal squamous cell carcinoma staging system for improved survival stratification. Oncotarget.

[16] Rice TW, Blackstone EH (2013). Esophageal cancer staging: past, present, and future. Thorac Surg Clin.

[17] Fujita H, Sueyoshi S, Tanaka T, Fujii T, Toh U, Mine T, Sasahara H, Sudo T, Matono S, Yamana H, Shirouzu K (2003). Optimal lymphadenectomy for squamous cell carcinoma in the thoracic esophagus: comparing the short- and long-term outcome among the four types of lymphadenectomy. World J Surg.

[18] Taniyama Y, Nakamura T, Mitamura A, Teshima J, Katsura K, Abe S, Nakano T, Kamei T, Miyata G, Ouchi N (2013). A strategy for supraclavicular lymph node dissection using recurrent laryngeal nerve lymph node status in thoracic esophageal squamous cell carcinoma. Ann Thorac Surg.

[19] Liu H, Lu L, Zhu Q, Hao Y, Mo Y, Liu M, Hu Y, Cui N, Rong T (2011). Cervical nodal metastases of unresectable thoracic esophageal squamous cell carcinoma: characteristics of long-term survivors after concurrent chemoradiotherapy. Radiother Oncol.

[20] Zhang P, Xi M, Zhao L, Li QQ, He L, Liu S, Shen J, Liu MZ (2014). Unilateral cervical nodal metastasis is an independent prognostic factor for esophageal squamous cell carcinoma patients undergoing chemoradiotherapy: a retrospective study. PLoS One.

[21] Zhang P, Xi M, Zhao L, Li QQ, He LR, Liu SL, Shen JX, Liu MZ (2014). Efficacy and prognostic analysis of chemoradiotherapy in patients with thoracic esophageal squamous carcinoma with cervical lymph nodal metastasis alone. Radiat Oncol.

[22] Yamashita M, Takenaka HY, Nakagawa K (2014). Semi-radical chemoradiotherapy for 53 esophageal squamous cell carcinomas with supraclavicular lymph node metastasis in a single institutional retrospective study. Hepatogastroenterology.

[23] Sheng L, Ji Y, Wu Q, Du X (2017). Regional hyperthermia combined with radiotherapy for esophageal squamous cell carcinoma with supraclavicular lymph node metastasis. Oncotarget.

[24] Seto Y, Fukuda T, Yamada K, Matsubara T, Hiki N, Fukunaga T, Oyama S, Yamaguchi T, Nakajima T, Kato Y (2008). Celiac lymph nodes: distant or regional for thoracic esophageal carcinoma?. Dis Esophagus.

[25] Amini A, Xiao L, Allen PK, Suzuki A, Hayashi Y, Liao Z, Hofstetter W, Crane C, Komaki R, Bhutani MS, Lee JH, Ajani JA, Welsh J (2012). Celiac node failure patterns after definitive chemoradiation for esophageal cancer in the modern era. Int J Radiat Oncol Biol Phys.

[26] Ma X, Li B, Yang S, Guo W, Zhu X, Li H, Xiang J, Zhang Y, Chen H (2014). Extent of lymph node dissection: common hepatic artery lymph node dissection can be omitted for esophageal squamous cell carcinoma. J Thorac Dis.

[27] Liu Q, Cai XW, Wu B, Zhu ZF, Chen HQ, Fu XL (2014). Patterns of failure after radical surgery among patients with thoracic esophageal squamous cell carcinoma: implications for the clinical target volume design of postoperative radiotherapy. PLoS One.

[28] Schomas DA, Quevedo JF, Donahue JM, Nichols FC, Romero Y, Miller RC (2010). The prognostic importance of pathologically involved celiac node metastases in node-positive patients with carcinoma of the distal esophagus or gastroesophageal junction: a surgical series from the Mayo Clinic. Dis Esophagus.

[29] Trovo M, Bradley J, El Naqa I, Foster E, Meyers B, Govindan R, Patterson A (2008). Esophageal carcinoma with celiac nodal metastases; curative or palliative?. J Thorac Oncol.

[30] Suh YG, Lee IJ, Koom WS, Cha J, Lee JY, Kim SK, Lee CG (2014). High-dose versus standard-dose radiotherapy with concurrent chemotherapy in stages II-III esophageal cancer. Jpn J Clin Oncol.

[31] Hironaka S, Ohtsu A, Boku N, Muto M, Nagashima F, Saito H, Yoshida S, Nishimura M, Haruno M, Ishikura S, Ogino T, Yamamoto S, Ochiai A (2003). Nonrandomized comparison between definitive chemoradiotherapy and radical surgery in patients with T(2-3)N(any) M(0) squamous cell carcinoma of the esophagus. Int J Radiat Oncol Biol Phys.

[32] Li G, Hu W, Wang J, Deng X, Zhang P, Zhang X, Xie C, Wu S (2010). Phase II study of concurrent chemoradiation in combination with erlotinib for locally advanced esophageal carcinoma. Int J Radiat Oncol Biol Phys.

[33] Minsky BD, Pajak TF, Ginsberg RJ, Pisansky TM, Martenson J, Komaki R, Okawara G, Rosenthal SA, Kelsen DP (2002). INT 0123 (Radiation Therapy Oncology Group 94-05) phase III trial of combined-modality therapy for esophageal cancer: high-dose versus standard-dose radiation therapy. J Clin Oncol.

[34] Willett CG (2002). Radiation dose escalation in combined-modality therapy for esophageal cancer. J Clin Oncol.

